# Risk Factors Contributing to the Occurrence and Recurrence of Hepatocellular Carcinoma in Hepatitis C Virus Patients Treated with Direct-Acting Antivirals

**DOI:** 10.3390/biomedicines8060175

**Published:** 2020-06-25

**Authors:** Sara Kishta, Ashraf Tabll, Tea Omanovic Kolaric, Robert Smolic, Martina Smolic

**Affiliations:** 1 Microbial Biotechnology Department, Genetic Engineering and Biotechnology Research Division, National Research Centre, El Behooth Street, Dokki 12622, Egypt; Sara.Mohamed@pei.de (S.K.); Ashraftabll@yahoo.com (A.T.); 2 Virology Division, Federal Institute for Vaccines and Biomedicines, Paul-Ehrlich-Institute, Paul-Ehrlich-Straße 51-59, 63225 Langen, Germany; 3 Department of immunology, Egypt Center for Research and Regenerative Medicine (ECRRM), Cairo 11517, Egypt; 4 Faculty of Medicine Osijek, Josip Juraj Strossmayer University of Osijek, J. Huttlera 4, HR-31000 Osijek, Croatia; tomanovic@mefos.hr (T.O.K.); rsmolic@mefos.hr (R.S.); 5 Faculty of Dental Medicine and Health Osijek, Josip Juraj Strossmayer University of Osijek, Crkvena 21, HR-3100 Osijek, Croatia; 6 Division of Gastroenterology/Hepatology, Department of Medicine, University Hospital Osijek, J. Huttlera 4, HR-3100 Osijek, Croatia

**Keywords:** hepatitis C virus, HCV, direct-acting antivirals, DAA, hepatocellular carcinoma, HCC, risk factors, occult HCV, liver cirrhosis, DNA ploidy, hepatitis B virus, HBV, non-alcoholic fatty liver disease, NAFLD

## Abstract

Although hepatitis C virus (HCV) RNA may be eliminated from blood circulation by direct-acting antivirals (DAA) therapy as assessed by real-time polymerase chain reaction (PCR), HCV RNA can still be present in liver tissue, and this is known as occult HCV. There has been a lot of controversy surrounding the recurrence of hepatocellular carcinoma (HCC) after DAA treatment of hepatic cells infected with chronic HCV. One of the main risk factors that leads to de novo HCC is the chronicity of HCV in hepatic cells. There are many studies regarding the progression of HCV-infected hepatic cells to HCC. However, there is a lack of research on the different molecular mechanisms that lead to the progression of chronic HCV infection to HCC, as well as on the effect of HCV on the alteration of DNA ploidy, which eventually leads to a recurrence of HCC after DAA treatment. In this review article, we will address some risk factors that could lead to the development/recurrence of HCC after treatment of HCV with DAA therapy, such as the role of liver cirrhosis, the alteration of DNA ploidy, the reactivation of hepatitis B virus (HBV), the role of cytokines and the alteration of the immune system, concomitant non- alcoholic fatty liver disease (NAFLD), obesity, alcohol consumption and also occult HCV infection/co-infection. Clinicians should be cautious considering that full eradication of hepatocarcinogenesis cannot be successfully accomplished by anti-HCV treatment alone.

## 1. Introduction

**Figure 1:**
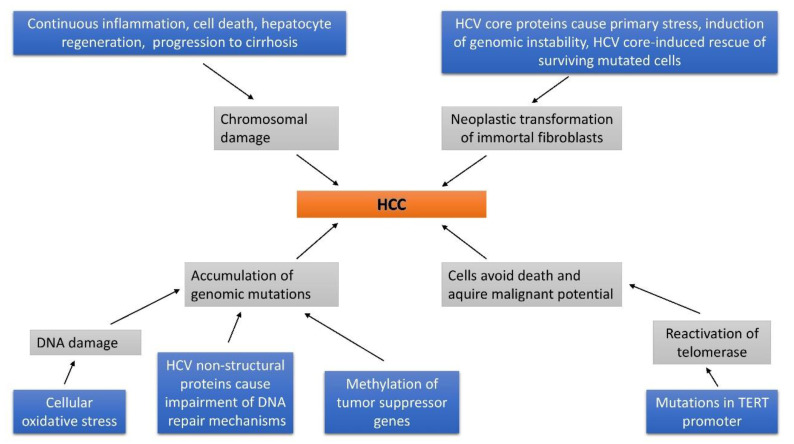
Development of hepatocellular carcinoma in hepatitis C virus (HCV) -infected individuals.

Hepatitis C virus (HCV) is a single-stranded RNA virus that targets hepatocytes and a member of the Flaviviridae family [1]. HCV represents one of the most common causes of chronic liver disease and, therefore, represents a major global public health problem [2]. HCV causes debilitating liver disease, which may progress to cirrhosis and cancer, and claims 500,000 lives annually worldwide [3]. Although the understanding of the HCV infection’s molecular virology has been greatly improved, the exact molecular mechanisms of disease progression leading to fibrosis, cirrhosis and hepatocellular carcinoma (HCC) are not completely resolved. Numerous clinical and experimental studies have demonstrated that HCV is capable of inducing hepatocarcinogenesis directly by its transcripts or proteins, and/or indirectly through induction of chronic liver inflammation [4]. Most of the infected patients are incapable of clearing the infection, consequently developing a chronic HCV infection [5]. HCV-related cirrhosis increases the risk for HCC. The frequency of HCC following treatment with direct-acting antivirals (DAA) agents is significant (3.3%) even after achieving sustained viral response (SVR), as reported by Aziz B et al. [6]. Many patients continue to have HCV-related disease progression after treatment with DAA therapy [7]. Although viral clearance in HCV-infected patients causes inhibition of further direct oncogenic interference, the reversal in the fibrosis level and the regenerative process are likely insignificant. Various authors consider the sudden decrease in viral load achieved with DAAs as a cause of distortion in the immune system [8]. As previously mentioned, a multistep process of HCV-induced hepatocarcinogenesis consists of a combination of pathway alterations caused either directly by viral factors or by the effect of immune mediators as a consequence of chronic inflammation, as shown in Figure 1 [2]. 

The WHO pronounced that 71 million people worldwide suffer from chronic HCV infection. A higher predominance of HCV infection is estimated in North Africa, the Middle East and Central and East Asia (>3.5%) [9], whereas it is lower in European (1.5%) and Eastern Mediterranean (2.3%) nations. However, about half a million deaths occur each year due to complications of HCV infection worldwide [10,11]. Egypt is considered the country with the world’s highest prevalence of HCV infection [12]. That is, nearly 13 million Egyptians are currently infected with HCV. HCV infection has an estimated national prevalence of 4% in the population aged 1–59 years in Egypt [13]. The predominance of HCV antibodies and HCV RNA among a 15–59-year old cohort was estimated to be 14.7 and 9.8%, respectively, according to the Egyptian Demographic Survey (EDHS) in 2008 on a large national sample. In other words, over 6.8 million persons aged 15–59 were positive for HCV antibodies, of which in more than 4.5 million individuals active HCV infection was present [13]. However, the prevalence of HCV infection among young Egyptian generations (newcomer students, Kafr Elsheikh University, north of Egypt) had markedly declined, with only 0.0028% of 9049 students positive for anti-HCV antibodies [14]. It is important to emphasize that the prevalence of HCV RNA and HCV antibody positive individuals were found to vary among governorates. Lower Egypt governorates, mostly rural in nature, still demonstrate a higher prevalence of HCV RNA and HCV antibodies in comparison to urban governorates [13]. Additionally, this study showed a notable reduction in the overall prevalence of HCV RNA positive individuals from 9.9 to 7.0% and HCV antibody positive individuals from 14.7 to 10% in the population aged 15–59, in the period of time between 2008 and 2015 [13]. The most important cause of this apparent significant HCV prevalence reduction is the fact that the group of individuals who were infected during the massive schistosomiasis treatment campaign with reused, poorly sterilized syringes (1960s through early 1980s) exceeded the age covered by the survey (i.e., those older than 59 years) [15]. Despite its high prevalence and highly infectious nature, HCV remains under-diagnosed and under-reported in most African countries [16]. HCC is the most common type of primary liver malignancy [17]. Additionally, HCC, as a predominant form of primary liver cancer, represents the third most common cause of cancer death worldwide [18]. The sudden increase in HCC incidence is attributed to the rise of the HCV infection incidence. Liver cancer is an exceptionally lethal disease, with a five-year survival rate below 20% [19]. Constant variations in HCC incidence are found by geographical area and ancestry. Namely the HCC incidence in Asian American community in the U.S. tripled between 1975 and 2005 [20]. Furthermore, some African and Asian countries show exceedingly high rates, with the highest one found in Mongolian men at 116.6 per 100,000 persons yearly, as opposed to 3.8 per 100,000 persons yearly in Northern European countries [21]. Geographical distribution of HCC alters considerably throughout the world with an incidence rate ranging from 2.1 in Central America to 35.5 in Eastern Asia [9]. HCV and HBV infection represent the most common cause of HCC. Their genomes integrate into the host’s DNA and consequently generate mutations. Less common causes of HCC are excessive alcohol consumption, contact with or consumption of aflatoxins and numerous metabolic disorders [22]. However, Waziry et al. [23] concluded that the best choice strategy for reduction in HCV-HCC incidence and mortality is the prevention of HCV transmission along with the prevention of liver disease progression among individuals with chronic HCV infection.

## 2. Possible Risk Factors for HCC Development

Chronic inflammation increases the levels of reactive oxygen species (ROS), followed by the hepatocyte damaging both at the genetic and metabolic level, eventually causing cell death. The HCV-altered environment enhances compensative liver regeneration which may favor chromosomal instability and irreversible genetic changes [24]. These changes are capable of promoting hepatocyte neoplastic transformation and the progression of malignant clones [24]. In this mini-review, we will focus on the four main risk factors for HCC progression and the potential recurrence (?) of HCC after DAA therapy. 

HBV infection is one of the most common causes of chronic liver disease and HCC. Reactivation of HBV after or during the clearance of HCV infection is a problem to consider, and more studies are required to determine the best management of this virological and clinical event, as reported by Pisaturo et al. [25]. Recently, Huang and Yu reported that HBV reactivation after or during HCV therapy has raised the possibility of using the prophylactic antivirals against HBV during DAA therapy [26]. Moreover, they added that in the post-SVR period, HCC still develops in a large proportion of patients, particularly among those with existing fibrosis and subjects who have ongoing risk factors (e.g., HBV dual infection, alcoholism, diabetes) [26]. In the DAA treated HCV infection, SVR is very high, but full attention must be given to the potential HCC existence as well as the HBV reactivation in patients with HBV/HCV co-infection who achieved SVR. The HCC existence was more often detected in patients with a past history of HCC [27]. The most reports about HBV reactivation (high ALT and HBV DNA levels) were made at 4–12 weeks of DAA therapy [28]. This study illustrated that the risk of HBV reactivation in these patients shows up to be low, which indicates that DAA treatment appears to be well tolerated in cancer patients with HBV/HCV co-infection [28]. However, most individuals with chronic or occult HBV infection are not conscious that they are infected. So, the risk of HBV reactivation rises significantly when HBV-infected individuals receive immunosuppressive or anticancer therapy [29].

It is estimated that by 2030 almost half of the world’s population will be suffering from obesity. There is also an increasing prevalence of non-alcoholic fatty liver disease (NAFLD), one of the inevitable sequelae of metabolic syndrome associated with obesity. Therefore, obesity and NAFLD play increasingly important roles in the epidemiology of HCC. Furthermore, various specific obstacles for proper HCC diagnosis, treatment and follow-up may occur in patients with liver steatosis. Despite the fact that there is a proven correlation between cirrhotic-NAFLD and high risk of HCC, the same correlation is not as clear for non-cirrhotic NAFLD. However, a rising number of studies report HCC even in patients with non-cirrhotic NAFLD. Mittal et al. [30] demonstrated, in a retrospective cohort study, that among 1500 United States veterans, nearly 13% of HCC occurred in patients without cirrhosis, with NAFLD being the main risk factor. It has also been proposed that in the group of patients suffering from HCC, the mortality rates are higher in patients with non-cirrhotic NAFLD as compared to patients with cirrhotic NAFLD [30].

### 2.1. Risk of DNA Methylation in the Progression of HCC

HBV and HCV are capable of inducing time-dependent, genome-wide changes in DNA methylation in infected mice with humanized livers [31]. Numerous methylated tumor suppressor genes were found in clinical specimens of HCV-infected liver tissue. Methylated tumor suppressor gene promoters are more often found in HCV-positive liver tissue as compared to HBV-positive or hepatitis virus-negative liver tissue [32]. Additionally, methylated tumor suppressor gene promoters are more common in liver cirrhosis as compared to chronic hepatitis [33]. These discoveries indicate that the methylation process is being accelerated by the chronic HCV infection. Methylation events were classified into three patterns: events showing significant differences between early HCC and non-cancerous liver, events showing a continuous increase with tumor progression, and methylation detectable only in advanced tumors [34]. Hence, the patterns of these methylation events have enormous variety [34]. Given their clinical relevance, numerous methylated tumor suppressor genes in the HCV-infected liver are positively correlated with time-to-HCC occurrence [34,35]. Toraih et al. [35] concluded that circulatory metastasis-associated lung adenocarcinoma transcript 1 (MALAT1) could be a presumptive non-invasive prognostic biomarker indicating worse liver failure score in HCV-related HCC patients along with traditional markers. It was found that DNA methylation alterations, which are not present in normal liver, can be found even in liver tissue affected with chronic hepatitis or cirrhosis associated with HBV or HCV infection, which are generally considered to be precancerous conditions for HCC [36].

### 2.2. Occult HCV

**Figure 2:**
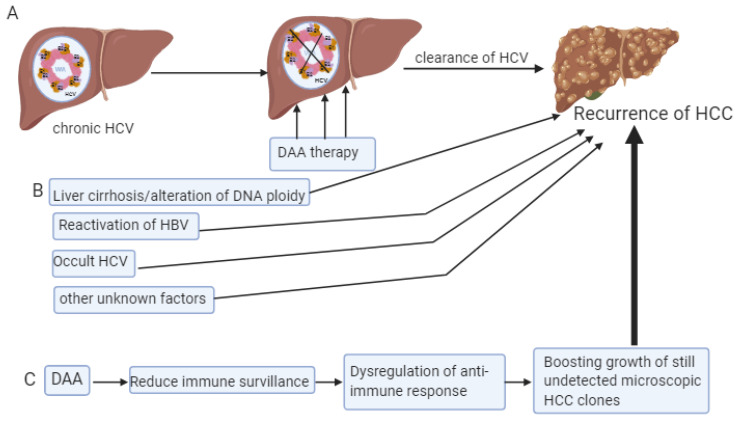
Schematic diagram representing the possibilities of HCC development in patients treated with direct acting antivirus agents (DAA). Panel (**A**) shows the development of HCC in patients treated by DAA. Panel (**B**) represents factors which may be associated with the development of HCC in general. Panel (**C**) represents a schematic hypothesis of HCC development in patients treated with DAA.

Occult HCV infection (OCI) is defined as the presence of HCV RNA in hepatocytes or peripheral blood mononuclear cells (PBMCs) with no detectable HCV RNA in the serum [37,38]. The existence of occult HCV is controversial. Indeed, some authors [38,39] could still detect RNA in hepatocytes and PBMCs and it is questionable whether these were intact viruses and if they were still able to replicate and cause damage or possibly even cause reactivation of HCV disease. Yousif et al. [38] found that the prevalence of OCI following treatment with DAAs was considerably high. Additionally, they recommended dual testing for HCV RNA which would be done in both PBMCs and serum in two time points- at the end of treatment of HCV infection with DAAs and during validation of the sustained viral response (SVR), following the initial response. In spite of successful viral clearance, numerous patients continue to exhibit HCV-related disease progression [39,40,41,42]. New DAAs are capable of eradicating HCV infection in more than 90% of patients with advanced liver disease. Despite that fact, liver cancer occurrence is not diminished even in efficiently treated cirrhotic patients who achieved SVR. Additionally, despite DAA treatment, a high risk of tumor recurrence in the short term is still present in patients previously treated for HCC [43]. Although IFN-free regimens eradicate HCV more efficiently, DAAs are capable of eliminating HCV from the serum, but not from the cells. Consequently, the replication of neoplastic clones still continues in the setting of reduced inflammation [44]. Accordingly, one may hypothesize that exogenous interferon (IFN) used as part of HCV therapy has a protective effect in HCC, because it activates inflammatory cells with pro-apoptotic and anti-cancer activities. In Serti et al.’s study from 2013, it was suggested that IFN-based treatment could have been responsible for the delay in HCC recurrence [45]. Therefore, Serti et al. [45] hypothesize that after HCV eradication with DAAs, the introduction of IFN into treatment could “re-activate” the immune response against neoplastic clones, promoting anti-cancer immunity (Figure 2). Although various clinical studies demonstrated that eradication of the hepatitis virus with IFN-based treatment results in the suppression of hepatocarcinogenesis, HCC may still develop after eradication of the virus [46,47]. Indeed, the five-year incidence of HCC after the SVR achieved with IFN-based treatment for HCV-related chronic liver disease is 2.3–8.8% [48]. Emphasis should also be put on the elderly and those with cirrhosis, due to the risk of HCC after achieving SVR. This fact could represent one of the most important issues in HCV-related hepatocarcinogenesis in upcoming years [49,50].

### 2.3. Immunity and Cytokines

Human immune system plays a key role in both viral clearance during acute infection and in liver damage during persistent hepatitis C. Due to the need for a cytoprotective effect, both innate and adaptive immune responses are important for the achievement of recovery from viral infection [51].

#### 2.3.1. HCV 

In chronic hepatitis C, HCV successfully avoids both innate and adaptive immune mechanisms [51]. The virus evades the host immune response by releasing a number of mediators, resulting in impaired virus clearance. [52] The latter is a result of CD4+ and CD8+ T cell dysfunction with impaired cytokine production and inadequate response to antigen stimulation [51,52]. In chronic HCV infection, a limited Th1-response and a predominant Th2 reaction have been described, whereas during acute self-limiting HCV infection, Th1 prevalence with circulating Helper T-cells that produce IFNα is proposed [53,54]. Barjon et al. [55] reported that effector T cells become exhausted over time and demonstrate an impaired antiviral activity, while CD4+ regulatory T cells (Tregs) accumulate in the liver during chronic HCV infection and are considered to be included in immune surveillance of tumors. Natural killer (NK) cells represent the model of innate lymphoid cells with antiviral properties and immune surveillance against cancer [56].

Natural killer (NK) cells are the model of innate lymphoid cells with antiviral properties and immune surveillance against cancer [56]. NK cells play an important role in the interferon innate production in the liver and their proportion increases during viral hepatitis infection [57] Additionally, there is a considerable association between NK and immune surveillance of cancer [58]. The levels of IL-2, IL-4, TNFα are more elevated than IFN-α and IL-10 in patients with chronic HCV [55]. However, IL-10 is produced by T cells, B cells, and macrophages and plays a key role in immunoregulation, as reported by Villani et al. [57]. Caja et al. suggested that several cytokines such as IL-6, IL-10, TNFα may be associated with cancer promotion and progression [59].

#### 2.3.2. HBV

Persistent necroinflammation and hepatocellular regeneration are major factors in the process of hepatocarcinogenesis, in addition to viral DNA and the oncoprotein HBx integration [60]. It is considered that HBV causes chronic liver damage through the activation of abnormal immune response [61]. Nevertheless, various changes occur in adaptive immunity due to the chronic HBV infection, all of which are potential immune pathogenic factors responsible for HCC development: immune tolerance, progressive immune activation, inactivation, reactivation and exhaustion. Chen and Tian et al. [61] reported that during chronic HBV infection, numerous immune cells participate in HCC development, such as: NK/NKT, HBV-specific CD8+ T cells, CD4+T, B, HBV-non-specific CD8+, and Kupffer cells.

### 2.4. Liver Cirrhosis and DNA Alteration

#### 2.4.1. HBV

As aforementioned, HBV is capable of contributing to HCC development through direct and indirect mechanisms. Both genomic instability and direct insertional mutagenesis of diverse cancer-related genes are induced by the integration of HBV DNA into the host genome at the early steps of clonal tumor expansion [62]. Altered versions of the preS/S envelope proteins and/or prolonged expression of HBx, which are recruited on the cellular chromatin and modulate chromatin dynamics at the specific gene loci, sensitizes liver cells to carcinogenic factors by dysregulating cell transcription and proliferation control. Activation of the unfolded proteins response, which may finally contribute to the hepatocyte transformation, is caused by the accumulation of preS1 large envelope proteins and/or preS2/S mutant proteins. Epigenetic changes targeting the expression of tumor suppressor genes occur early in the HCC development. HBV-related HCC, as compared to HCCs associated with other risk factors, have a higher rate of p53 inactivated by mutations, overexpression of fetal hepatic progenitor cells genes and chromosomal alterations. HBV-related HCCs may occur in the non-cirrhotic liver, further supporting the consideration that HBV plays a direct role in liver transformation through several mechanisms, such as stimulating the host immune response and driving liver chronic necro-inflammation and also triggering both common and etiology specific oncogenic pathways [62,63].

Furthermore, it was reported that HCC associated with a previous or actual HBV infection possesses a peculiar appearance, characterized by increasing ploidy and reduction of binocularity [64]. However, when HBV and HCV are identified as causative viruses of post-transfusion hepatitis, they evolve into persistent infections, consequently leading to the progression of chronic hepatitis to cirrhosis, and HCC. Unlike other hepatic viruses, HBV is difficult to be eradicated completely. Despite the fact that vaccination can prevent HBV infection, reactivation of HBV following immunosuppressive therapy and anti-cancer chemotherapy represents a well-known complication. HBV reactivation has already been associated with the TNF-α inhibitor-containing immunosuppressive therapy and anti-CD20 monoclonal antibody rituximab-containing chemotherapy in HBV-resolved patient [22]. In addition, it was reported that postoperative HBV reactivation (PHR) is associated with resection-induced immunosuppression in patients with HBV-related HCC [65]. Controlling Nutritional Status (CONUT) score represents an efficient index for evaluating immune-nutrition function [65]. However, its value as the predictor of PHR in HBV-HCC patients remains unclear. Moreover, HBV reactivation after curative resection is the cause of HBV-related HCC recurrence in patients with a low viral load [66].

#### 2.4.2. HCV

The vast majority of liver cancers were identified at early stages in one large, single-center cohort of patients with cirrhotic liver who received DAA treatment for HCV infection [67]. Various factors, such as diabetes, male sex and markers of liver fibrosis can be used to identify patients at increased risk for HCC following DAAs therapy [67]. Diagnosis of HCC using cell cycle analysis (DNA ploidy) is helpful in the detection of cellular and structural abnormalities. Modern technologies such as flow cytometry and image analysis for the determination of changes in DNA content in human cancer cells are among the most often used ways to diagnose and predict neoplasms, as reported by several studies [68,69]. Moreover, liver cell dysplasia is associated with aneuploidy and elevated DNA index and carries the same risk factor for the development of hepatocellular carcinoma, as reported in our previous study [70]. The potential benefit of image cytometry in the evaluation of the different histopathological problems has been confirmed [71,72]. Moreover, various studies have demonstrated a strong correlation between the analysis of DNA content by flow cytometry and the computer-based image technique [69,73,74]. Studies indicate that, currently, the major cause of liver-related death in patients with compensated cirrhosis is HCC. 

Almost all HCC cases occur in the presence of advanced fibrosis or cirrhosis. Consequently, any cause of liver disease that may result in advanced fibrosis or cirrhosis should be considered as a potential risk factor for HCC development. Alterations in DNA content that could lead to liver disease progression, malignant transformation and development of HCC have been found in HCV positive liver tissue [75]. Ke et al. [76] reported that HCV possesses the ability to activate numerous cellular responses, which consequently could participate in the pathophysiology of HCV-related liver disease through changes in lipid metabolism, interference with cell growth and/or cell proliferation and stimulation of the oncogenic signal pathway. However, the mechanism of HCC caused by HCV still remains unclear. 

In order to clarify our point, we focused on three main groups as shown in Table 1.

## 3. Patients with HCV, HBV Hepatitis and Without Liver Diseases Progression

Machida et al. [77] suggested that HCV infection may inhibit the mitotic checkpoint, therefore inducing polyploidy, which can further lead to neoplastic transformation. Data of Smirnova et al. [79] suggests that HCV core proteins may induce neoplastic transformation through various mechanisms, such as induction of genomic instability, primary stress and HCV core-induced preservation of surviving mutated cells [79]. However, Suhail et al. [80] recently reported that there is a firm correlation between core, host single nucleotide polymorphisms (SNPs), HCV variants within NS4, NS5A and the pathogenesis of HCC. In our previous study, we investigated the alteration of the total DNA content in liver tissues of different histopathological grade. The image cytometry analysis was conducted in HCV positive and negative tissues. We assessed cellular kinetics in fibrotic and cirrhotic liver, and malignant hepatoma in HCV infected patients and compared their content to the positivity of HCV RNA [75]. Moreover, HCV RNA was measured in serum and liver tissue samples. The results demonstrated that 50% of patients were positive for HCV RNA in their sera samples while 69% had detectable HCV RNA in liver tissue [75]. The explanation of variation of detectable HCV viremia in sera samples and tissues is not clear, but it may be due to the activity of the immune system in peripheral blood and/or virus replication rate being higher in hepatocytes than in the peripheral blood [81,82]. HCV infection promotes the multi-step process of hepatocarcinogenesis by inducing continuous inflammation, hepatocytes necrosis and regeneration and progressive liver cirrhosis. All the results confirmed that HCV positive liver tissue had increased DNA content which may lead to malignant transformation of the liver cells and progression to cancer. These results can be explained by the fact that HCV induces strong cellular oxidative stress, which derives DNA damage events and provokes accumulation of genomic mutations. Moreover, impaired DNA repair mechanisms due to interaction with HCV non-structural proteins have been proposed to be the cause of HCC development in chronic HCV [83,84,85]. DNA abnormalities and pro-mutagenic DNA lesions were reported to be more frequent in chronic HCV-infected patients [86]. Thus, our data showed that HCV has a role in the progression of liver disease and HCC. However, further study with a greater number of liver tissue specimens is required, particularly for HCV-negative HCC, and also measurement of DNA ploidy and S phase proliferation by flow cytometry would be preferable to confirm the results of image cytometry analysis. Furthermore, the results of Sanad et al. [71] demonstrated increased S-phase fractions’ values in chronic hepatitis and cirrhotic patients.

## 4. Patients with HCV or/and HBV Hepatitis with Liver Diseases Progression

Gomaa et al. [87] reported that continuous inflammation, cell death, hepatocyte regeneration within chronic hepatitis and consequently progression to cirrhosis are considered to lead to chromosomal damage and to initiate hepatic carcinogenesis. Gehrau et al. [88] suggested that severe inflammation in the non-cancerous HCV cirrhotic liver section might accelerate the promotion to HCC in HCV infected patients. Recently, it was documented that abnormal liver function seen in HCV patients may be explained by the fact that oncogenesis in HCV patients always appears in a cirrhotic liver, which develops from the fibrosis, indicating the greater extent of liver damage happening in HCV patients [89]. Furthermore, the results of Toyoda et al. [50] demonstrated that the percentage of mononuclear polyploid hepatocytes increased in patients with high hepatitis activity (HBV and HCV) and marked fibrosis. Smirnova et al. [79] showed that the long-term HCV core expression alone is sufficient for the complete transformation of immortal fibroblasts that can afterward induce cancer in a susceptible host. HCV infection is associated not only with the development of HCC, but probably also with non-Hodgkin’s B-cell lymphoma [90]. Werling et al. [78] showed that by maintaining liver inflammation and hepatocyte necrosis and regeneration, HCV induces chronic hepatitis and cirrhosis, but also acts as a cofactor in hepatocarcinogenesis.

## 5. Patients with Non-Viral Hepatitis with Liver Diseases Progression 

Cyclin A protein expression and cytometrically measured S-phase fraction were found to be significantly lower in chronic HCV infection as compared to chronic non-C hepatitis, therefore representing decreased cell proliferation of virus-infected cells. In the chronic HCV group, cyclin A protein expression and the grade of inflammation were decreased, and the S-phase fraction depression was moderate, mainly in the severe grade group [78]. DNA content was measured in liver tissues of several histopathological grades by computer-based image cytometry. The results showed a higher degree of alteration in DNA ploidy: (more than G0/G1) 80% in hepatocellular carcinoma patients, compared to 40% in cirrhotic patients, 20% in fibrotic patients and 4% in normal liver tissues (more than G0/G1). Since all HCC patients were infected with HCV, the results also showed that in HCV infected group more than 80% of the cells were in the tetraploidy and aneuploidy regions. This indicates the high degree of alteration in DNA ploidy in HCV positive patients as compared to HCV negative patients (negative HCV are a group of cirrhotic and fibrotic liver tissues without HCV infection). Moreover, the results of the histochemical analysis confirmed the DNA cytometry results and showed that carcinoma tissues had more DNA content than cirrhotic or fibrotic tissues with statistical significance (*p*-value < 0.05). On the other hand, the results of the histochemical analysis demonstrated that positive HCV RNA liver tissues had significantly more DNA content as compared to HCV RNA-negative liver tissues (*p*-value < 0.05). These results are in agreement with the findings of others [72]. DNA content of hepatocytes in patients with chronic HCV infection and patients with non-viral chronic hepatitis was studied by Werling et al. [78]. The results showed that chronic hepatitis C patients have a DNA index significantly higher than non-viral hepatitis patients [78]. It was reported that the presence of a HCV proliferation effect could be a possible explanation for this deviation.

## 6. Recurrence of HCC Despite the DAA Therapy

The latest improvements in the HCV infection treatments include the introduction of DAAs [91]. Despite the high effectiveness of DAA therapy in HCV treatment, DAAs still bear the risk of HCC development [91]. In HCV patients, the incidence of HCC decreases significantly after achieving SVR with DAAs [92]. In other words, new DAAs are capable of eradicating HCV infection in over 90% of patients with advanced liver disease. Unfortunately, the reduction in HCC incidence in successfully treated cirrhotic patients is not achieved. In addition, patients previously treated for HCC still have a high risk of tumor recurrence in the short term [57]. The data mentioned earlier in this article implicate that HCC risk reduction after HCV eradication could be achieved with IFN-based regimens. However, in transplanted patients this approach is not applicable because of the high risk of graft rejection as a consequence of IFN treatment. Recent progress in genomic analysis, such as whole genome sequencing, had a significant impact on the discovery of genetic and epigenetic aberrations responsible for the process of HCV-associated hepatocarcinogenesis. The accumulation of these genetic and/or epigenetic aberrations has already occurred in the liver tissue along with long-term HCV infection, indicating that chronically damaged liver has substantial cancerous potential, although the hepatitis virus is already eradicated or suppressed [93]. Despite the fact that eradication and/or suppression of the hepatitis virus may be partly responsible for the reduction in the HCC incidence, multicentric tumors often develop in patients even after the clearance of the infecting viruses [94].

## 7. Other Factors

Ethnic group, race or geographical regions are also responsible for the distribution of risk factors in patients with HCC. The various prevailing risk factors are: infection with HBC or HCV, alcoholic liver disease, NAFLD, aflatoxins, etc. [95]. Most of these risk factors facilitate the development and progression of cirrhosis which is the most common finding in HCC patients [96]. Another well-defined risk factor for HCC is continuous chronic alcohol abuse (40–60 g of alcohol per day) which is also capable of promoting the development of cirrhosis. HCV infected individuals, who are known for their heavy use of alcohol are more likely to develop HCC as compared to those who do not abuse alcohol [97]. Generally, all the risk factors transform normal liver to HCC through fibrosis and cirrhosis [95].

## 8. Conclusions

HCV has a substantial role in the development of HCC. HCV may be eliminated from blood circulation by DAA therapy as assessed by real-time PCR, but is still present in liver tissue and/or PBMCs (OCI). In addition, all physicians should be aware of the fact that anti-HCV therapy alone is not capable of fully preventing hepatocarcinogenesis and attention should be given to the various factors associated with hepatocarcinogenesis, such as occult HCV, HBV infection and liver cirrhosis/alteration of DNA ploidy. 

## Figures and Tables

**Table 1:** Liver cirrhosis/fibrosis with or without HBV/HCV infection.

		HBV/HCV Infection Without Cirrhosis/Fibrosis	HBV/HCV Infection With Cirrhosis/Fibrosis	Cirrhosis/Fibrosis Without HBV/HCV Infection
HCV	Core	mitotic checkpoint induces Polyploidy leading to neoplastic transformation [77]	cofactor-induced chronicity and cirrhosis [78]	-
		neoplastic transformation-driven Genomic instability, due to preservation of surviving mutated cells and initiation of primary stress [79]	long term expression of HCV core protein transforms immortal fibroblasts leading to HCC [79]	-
		firm correlation with SNP within NS and NS5A leads to HCC [80]	-	-
	RNA	50% positive sera and 69% positive in tissue	-	-
	DNA content	significant amount of HCV RNA in the liver without DNA ploidy [75]	-	Alteration in DNA ploidy: 80% in HCC, 0% in cirrhotic, 20% in fibrotic and 4% in normal participants [75]
		80% more tetra and aneuploidy as compared to HCV-negative-cirrhotic and fibrotic liver [75]	-	-
HBV and HCV		-	mononuclear DNA polyploidy in hepatocyte increases with fibrosis	-
S phase fraction		-	-	Increased in cirrhosis and chronic liver disease [71]
		-	-	significantly decreases in HCV compared to chronic non- HCV
Cyclin A		-	continuous inflammation	significant decrease in HCV than chronic non-HCV
		-	Causes chromosomal damage leading to HCC	
Inflammation		-	hepatocyte regeneration	-
		-	cell death	-
		-	Non-cancerous HCV cirrhotic accelerate HCC	-
